# Recycled Components in 3D Concrete Printing Mixes: A Review

**DOI:** 10.3390/ma18194517

**Published:** 2025-09-28

**Authors:** Marcin Maroszek, Magdalena Rudziewicz, Marek Hebda

**Affiliations:** Department of Materials Engineering, Faculty of Materials Engineering and Physics, Cracow University of Technology, Warszawska 24, 31-155 Kraków, Poland; marcin.maroszek@doktorant.pk.edu.pl (M.M.); magdalena.rudziewicz@doktorant.pk.edu.pl (M.R.)

**Keywords:** 3D concrete printing (3DCP), recycled construction materials, supplementary cementitious materials (SCM), sustainable construction, circular economy, carbon footprint, life cycle assessment (LCA)

## Abstract

Rapid population growth and accelerating urbanization are intensifying the demand for construction materials, particularly concrete, which is predominantly produced with Portland cement and natural aggregates. This reliance imposes substantial environmental burdens through resource depletion and greenhouse gas emissions. Within the framework of sustainable construction, recycled aggregates and industrial by-products such as fly ash, slags, crushed glass, and other secondary raw materials have emerged as viable substitutes in concrete production. At the same time, three-dimensional concrete printing (3DCP) offers opportunities to optimize material use and minimize waste, yet it requires tailored mix designs with controlled rheological and mechanical performance. This review synthesizes current knowledge on the use of recycled construction and demolition waste, industrial by-products, and geopolymers in concrete mixtures for 3D printing applications. Particular attention is given to pozzolanic activity, particle size effects, mechanical strength, rheology, thermal conductivity, and fire resistance of recycled-based composites. The environmental assessment is considered through life-cycle analysis (LCA), emphasizing carbon footprint reduction strategies enabled by recycled constituents and low-clinker formulations. The analysis demonstrates that recycled-based 3D printable concretes can maintain or enhance structural performance while mix-level (cradle-to-gate, A1–A3) LCAs of printable mixes report CO_2_ reductions typically in the range of ~20–50% depending on clinker substitution and recycled constituents—with up to ~48% for fine recycled aggregates when accompanied by cement reduction and up to ~62% for mixes with recycled concrete powder, subject to preserved printability. This work highlights both opportunities and challenges, outlining pathways for advancing durable, energy-efficient, and environmentally responsible 3D-printed construction materials.

## 1. Introduction

Rapid population growth and accelerating urbanization impose unprecedented demands on the construction industry, particularly regarding the supply of aggregates and concrete. Global aggregate consumption is projected to reach 50–55 billion tonnes by 2030 [1], exerting significant pressure on natural resources and the environment. Concrete, the most widely produced construction material worldwide, accounts for approximately 70% of aggregate mixtures [2], with production largely dependent on Portland cement [3]. Substituting virgin constituents with recycled ones has emerged as a viable pathway to lower embodied impacts while maintaining performance, chiefly by reducing extraction and transport burdens [4]. Concurrently, emerging technologies such as three-dimensional concrete printing (3DCP) provide additional opportunities for sustainable construction. 3DCP facilitates precise material deposition and significantly reduces waste generation; however, it requires the development of customized mix designs with optimized rheological and mechanical characteristics [5,6]. At the same time, new challenges have emerged, particularly those related to the thermal conductivity of printed layers and their implications for building thermal performance and occupant comfort. Recent advances demonstrate that 3DCP can facilitate the fabrication of wall systems with optimized internal geometries, allowing the integration of in-wall insulation and thereby enhancing the thermal performance of buildings [7,8,9].

This review synthesizes current evidence on the use of recycled materials in 3D concrete printing, highlighting how such approaches can reduce the environmental footprint of construction through printable binders and mortars aligned with circular-economy principles [10,11].

### Environmental Issues

**Figure 1:**
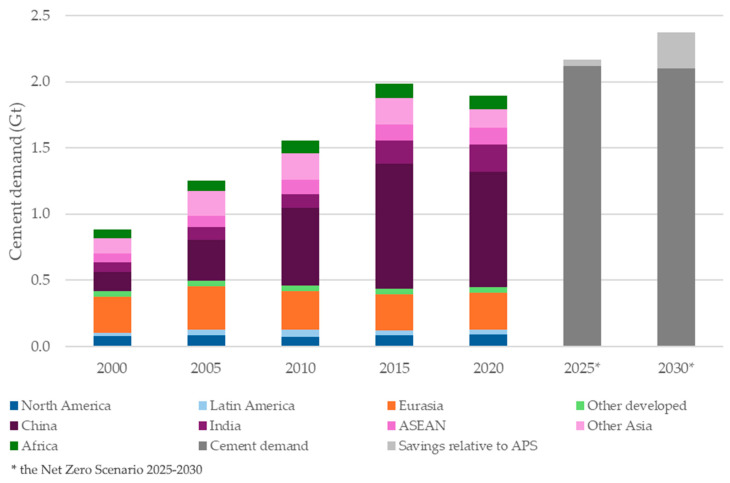
Global cement demand for building construction by region (2000–2020) and projections for 2025–2030 under the Net Zero Emissions (NZE) Scenario. The NZE Scenario (IEA) is a 1.5 °C-consistent pathway that reaches net-zero energy-sector CO_2_ emissions by 2050; it assumes strong material-efficiency measures, clinker substitution, recycling, and deployment of low-carbon cements, which lower demand relative to the Announced Pledges Scenario (APS). Dark-grey bars show total demand in NZE; light-grey segments indicate savings relative to AP (ASEAN—Association of Southeast Asian Nations) [26].

The built environment exerts large pressures on energy and material use, and EU policy (e.g., EPBD) continues to lower operational energy demand in buildings [12]. As operational loads decline, embodied impacts from materials become a major lever for mitigation at the product (cradle-to-gate) stage. In concrete, these impacts are driven primarily by cement production (clinker calcination), while aggregate extraction and processing contribute a smaller—but non-negligible—share [11,13,14,15,16,17]. Figure 1 summarizes global demand trends relevant to cement and aggregates, providing the macro context for material substitution.

**Figure 2:**
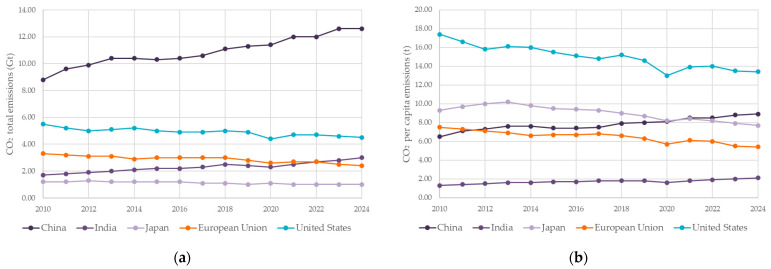
Carbon emissions trends varied widely across regions. CO_2_ total emissions and CO_2_ per capita emissions by region, 2000–2024: (**a**) CO_2_ emissions, (**b**) emissions per capita [27,28,29].

At mix level, Portland cement typically dominates the embodied CO_2_ of concrete (≈74–81%), with aggregates contributing ≈ 13–20% [18,19,20]. Reducing the clinker factor via supplementary cementitious materials and incorporating recycled constituents can therefore deliver meaningful, near-term reductions without sacrificing performance—especially when mixes are tailored for process windows specific to 3D concrete printing (3DCP). Figure 2 positions these material-level interventions within broader emission pathways, highlighting the importance of addressing product-stage impacts alongside operational efficiency. This review concentrates on that intersection: the role of recycled constituents in printable binders and mortars, their influence on fresh-state parameters and hardened/thermal performance, and consistent LCA reporting for cradle-to-gate assessments [13,20,21,22,23].

Despite rapid progress, evidence on recycled constituents for 3D concrete printing remains fragmented. Reporting of rheological properties relevant to printability is often inconsistent, with varying metrics and protocols for parameters such as yield-stress development, thixotropy, and open time. This lack of standardization complicates comparisons across studies. Similarly, links between mix design, interlayer bonding, and anisotropy—and their consequences for durability, transport performance, and fire or thermal resistance—are only partially established. Life-cycle assessments also show limited harmonization with respect to system boundaries, functional units, and the treatment of printing-stage energy and waste, which obscures a clear understanding of net environmental benefits. This review addresses these gaps by organizing rheology descriptors around printable windows, relating recycled constituents to directional and service performance of printed elements, and proposing a concise, 3DCP-specific LCA reporting checklist [10,24,25].

## 2. Recycled Concrete Additives

This section focuses on supplementary cementitious materials (SCMs) and waste-derived fine fractions relevant to 3D concrete printing (3DCP). Materials with pozzolanic or latent-hydraulic activity—including fly ash, bottom ash (when finely ground), slags, silica fume and metakaolin—can partially replace Portland cement with documented effects on rheology and strength development [30]. In 3DCP, mix design must satisfy process-critical rheological criteria—extrudability, filament shape retention and adequate open time—in addition to conventional mechanical targets [31]. Fine fractions of waste-derived materials, such as recycled fine aggregates (RFAs) or powders produced from the crushing of concrete, ceramics, or demolition bricks, play a particularly critical role in this regard. These constituents can function not only as substitutes for natural aggregates and mineral admixtures but also as modifiers of thixotropic behavior, thereby improving the printability of fresh mixtures.

Waste-derived fines are particularly impactful in this context. Recycled fine aggregates (RFAs) influence yield stress and open time, affecting pumpability and layer stability; excessive replacement generally compromises green strength and directional strength, even if buildability initially improves. By contrast, finely ground powders from crushed concrete/ceramics/bricks can enhance particle packing and structural build-up, which is advantageous for formwork-free printing where early stiffness governs print fidelity. The integration of such additives within 3DCP—complementary to geometry/material savings achievable by topology-optimized toolpaths—supports lower-clinker, resource-efficient mixes, provided rheology is tuned to the process window [32].

The following subsections characterize each recycled constituent and quantify its impact on fresh-state and hardened properties with respect to the specific performance requirements of 3DCP.

### 2.1. Addition of Fly Ash and Coal Bottom Ash

Fly ash (FA) is generated in coal-fired power plants during the combustion of coal, with substantial quantities historically stored in landfills, a practice associated with both environmental and economic drawbacks [33,34]. The incorporation of FA and silica fume (SF) as partial replacements for Portland cement reduces cement demand and thereby lowers the production cost of autoclaved aerated concrete (AAC) [35,36] while simultaneously improving selected performance properties [30,37]. FA particles are collected from flue gases by electrostatic precipitators, with particle size distribution depending on the collection point within the system. Material captured from downstream sections (e.g., rear hoppers) generally contains finer fractions. FA formation occurs when mineral impurities in coal, such as clay, feldspar, quartz, and shale, melt under combustion and subsequently solidify into spherical, glassy particles upon rapid cooling.

The properties of FA are influenced by several factors, including coal type, combustion conditions, and collection methods. Class F FA, derived from bituminous coal, is rich in silica, alumina, and iron but low in calcium, requiring activation with cement or lime. Class C FA, produced from lignite or sub-bituminous coal, contains higher calcium levels, facilitating accelerated strength development. Incorporating FA into concrete enhances workability, reduces drying shrinkage, and contributes to strength development (Table 1) while also providing a sustainable solution to the waste disposal problem. Due to its physical properties and availability, FA is also often used as a substitute for natural sand. [38,39]. Chemically, FA typically contains silicon dioxide (SiO_2_), aluminum oxide (Al_2_O_3_), iron oxide (Fe_2_O_3_), calcium oxide (CaO), and residual unburned carbon (approximately 90% of the remaining organic fraction). Its mineral composition includes crystalline phases (quartz, mullite, hematite) and amorphous glassy phases (silica glass) [40]. Owing to incomplete combustion and rapid cooling, FA particles adopt a predominantly spherical morphology [41,42]. A substantial fraction (65–90%) of FA particles are smaller than 0.01 mm, which, combined with their spherical shape, improves rheology, reduces interparticle friction, and facilitates a more homogeneous and easily pumpable mixture. For instance, Xu et al. [43] demonstrated that FA, due to its spherical morphology, initially reduces viscosity and shear stress, thereby lowering yield stress and improving extrudability. When FA content exceeds approximately 20%, the increased water demand reverses earlier improvements and reduces flowability. This change in rheology directly affects printability: extrusion length rose from 40 mm to 91 mm with higher FA contents, but excessive levels (>30–40%) led to loss of layer stability and weaker interlayer bonding. Buildability was highest at 20% FA, with minimal deformation and preserved interlayer integrity, whereas higher replacement levels compromised structural stability. At moderate additions (~10–15%), FA improves rheology through a lubrication effect that lowers yield stress and viscosity; however, at higher dosages (>20–25%), this advantage is offset by greater water demand, resulting in reduced filament stability and print accuracy [44].

Recent studies highlight the potential of nano-fly ash (N-FA) in 3DCP. Taqa et al. [45] demonstrated that incorporating 5–15% N-FA significantly enhanced workability, increasing flow spread by up to 41.3%, reducing extrusion pressure, and improving print stability, which allowed a greater number of layers to be deposited without deformation. In mixtures containing polyoxymethylene (POM) fibers [46], FA addition improved rheological performance: at 20% FA, dynamic yield stress decreased by 21.69–32.89% depending on fiber content (1.5–2.5%), while plastic viscosity decreased by 16.95–21.58%. These results confirm the “ball-bearing lubrication” effect of FA, which facilitates paste flow, improves fiber dispersion, and enhances overall workability. The most favorable balance between printability and buildability was observed at 10–15% N-FA. At 5% N-FA, compressive strength increased by 19.4% (printed specimens) and 25.9% (cast specimens), while flexural strength improved by 46.1% and 33.4%, respectively. The maximum flexural strength gain of 52.5% was recorded for 10% N-FA in printed mixtures [45]. Tseng et al. [47] found that FA enhanced flowability due to the spherical morphology of its particles, reducing interparticle friction and improving extrusion uniformity.

Other studies [48,49] further corroborate these findings, indicating that an FA content of approximately 10% provides optimal improvements in rheological properties without significant loss of mechanical performance. For example, mixtures with 10% FA achieved flexural and compressive strengths of 18.5 MPa and 144.6 MPa, respectively, compared to reference values of 17.9 MPa and 136.4 MPa [48]. Moreover, compressive strength at 10% FA was 37.5% higher than that of mixtures with 20% FA [49]. Collectively, these results confirm that FA, particularly in nano-scale form, offers significant potential for improving both fresh-state properties and hardened performance in 3D printable concretes.

**Table 1:** Properties of fly ash used in conventional and 3D printed concrete.

FA Class	% Replacement in Cement	Particles Size	Density	Compressive Strength (MPa)	Specimens Type	Reference
F	10, 30, 50%	-	-	23.02 MPa, 19.73 MPa, and 19.25 MPa, respectively	cast specimens	[50]
F	50%	<10 µm	2.2 g/cm^3^		cast specimens	[51]
F	25, 50%	-	2.08	46 MPa and 36 MPa, respectively	3D-printed specimens	[47]
HFA (high-temperature fly ash)	35%	<400 µm	2.41 g/cm^3^	40 MPa	3D-printed specimens	[52]
C	0%, 25%, 50%	<100 µm	-	29–40 MPa.	3D-printed specimens	[53]
F	5, 10, 15%	≤1 µm (nano-FA)	~2.20 g/cm^3^	Increase strength up to ~35 MPa after 28 days	3D-printed specimens	[45]
C	100%	-	-	32.63–34.86 (28 days)	3D-printed specimens	[48]

Coal bottom ash (CBA) is a by-product generated in conventional pulverized coal-fired boilers, where the heavier particles settle at the bottom of the combustion chamber. It is a porous, granular material with a dark coloration and a morphology resembling gravel or coarse sand. After drying and grinding (ground coal bottom ash, GCBA), it exhibits pronounced pozzolanic activity, which increases with particle fineness. In its dry state, CBA consists primarily of silica, alumina, iron oxides, and calcium oxide and may contain residual unburned carbon. The latter influences both the loss on ignition (LOI) and the water demand of mixtures containing CBA. Compared to coal slag, CBA demonstrates higher chemical reactivity, as it can react with calcium hydroxide generated during cement hydration to form additional calcium silicate hydrate (C–S–H) phases. This secondary hydration process enhances both the compressive strength and the impermeability of concrete. The literature suggests that fine coal bottom ash (CBA) improves pozzolanic reactivity and mechanical properties, but excessive fineness or high replacement levels can negatively impact rheology, increasing water demand and reducing mix stability [54].

According to [55], fly ash (FA) and bottom ash (CBA) originating from the same coal-fired power plant exhibit favorable compressive strength development and a chemical composition conducive to capillary pore refinement through the formation of reactive hydration products. This process leads to a modified pore size distribution, contributing to improved durability performance. The principal differences between FA and CBA are summarized in Table 2.

The utilization of coal bottom ash (CBA) in concrete technology has gained increasing attention owing to its economic benefits and environmental advantages. Ganesan et al. [57] evaluated CBA as a cost-effective fine aggregate replacement in combination with ultrafine slag material (Alccofine) as a partial cement substitute. The optimal mixture, comprising 40% CBA and 15% Alccofine, resulted in the highest compressive strength. Similarly, Chuang et al. [58] investigated the replacement of Portland cement with ground CBA (GCBA) in conjunction with fly ash (FA) derived from the same power plant. Their findings revealed that, at a water-to-binder ratio (W/B) of 0.5 and a 60% cement replacement by FA, compressive strength increased markedly from 11.00 MPa (7 days) to 40.75 MPa (180 days), demonstrating excellent long-term performance. For finely ground CBA (FGCBA), a 20% replacement level provided higher early (24.27 MPa) and final (44.10 MPa) strengths, while a 60% substitution achieved only modest improvements (9.49 to 33.02 MPa), indicating reduced efficiency of FGCBA at elevated dosages.

The pozzolanic potential of GCBA has been confirmed in multiple studies. The degree of grinding is critical: Oruji et al. [59] showed that GCBA with a median particle size of D50 ≈ 5 µm exhibits reactivity comparable to FA, while Guan et al. [60] demonstrated that finer particles improve mortar strength. Excessive grinding, however, can impair the rheological behavior of fresh mixes. Aydın [61] evaluated mortars with 25–70% CBA replacement and found that balanced substitution levels ensured satisfactory strength and durability. Similarly, Poudel et al. [62] reported optimal mechanical performance at moderate GCBA contents (10–20%). At higher replacement levels, additional measures such as nanomaterials or superplasticizers were required to maintain adequate properties. These distinctions are critical in determining the practical applications of FA and CBA. Fly ash, due to its high pozzolanic reactivity, is primarily employed as an active binder component in concrete mixtures. By contrast, the porous structure of CBA enhances water retention, but at high replacement levels it requires the addition of mix-modifying admixtures. The fundamental difference between the two materials lies in their chemical reactivity, very high for FA and moderate for GCBA, which dictates their suitability for specific applications. From an environmental standpoint, both materials present significant potential: FA reduces cement consumption and, consequently, CO_2_ emissions, whereas GCBA can serve not only as a cement substitute but also as a fine aggregate or a raw material for eco-friendly brick production, offering a sustainable alternative to traditional fired clay products [63].

### 2.2. Addition of Ground Granulated Blast Furnace Slag

Partial replacement of cement with ground granulated blast-furnace slag (GGBFS) represents one of the principal strategies for producing sustainable concrete, as it effectively reduces the environmental footprint of construction materials. GGBFS, a by-product of the steel industry, is widely recognized for its capacity to enhance concrete durability, improve resistance to chemically aggressive environments, and lower CO_2_ emissions. Substituting a portion of Portland cement with GGBFS not only reduces the demand for natural resources but also decreases the energy intensity of traditional cement production. As a result, slag-modified concrete offers a combination of environmental, mechanical, and durability benefits, making it a compelling alternative in sustainable construction practices.

**Figure 3:**
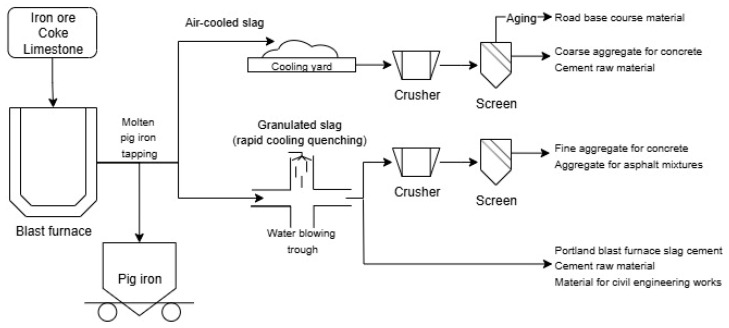
Production flow of the blast furnace slag [64].

GGBFS is produced during the manufacture of pig iron in blast furnaces (Figure 3). After tapping, the molten slag is routed either to an air-cooling yard or to a water-granulation trough. Slow air-cooling followed by aging, crushing and screening yields a largely crystalline air-cooled slag, typically used as road-base, coarse aggregate for concrete, or as cement raw meal. In contrast, rapid quenching with pressurised water produces a glassy granulated slag; when sieved it can serve as fine aggregate for concrete or asphalt, and when dried and ground it becomes ground granulated blast-furnace slag (GGBFS)—a latent-hydraulic and pozzolanic binder. Thus, the cooling route and point of collection control the properties and the end use of the slag. Due to these physico-chemical characteristics, GGBFS is extensively employed as a partial substitute for ordinary Portland cement (OPC), providing environmental, economic, and technological advantages. Notably, replacement of 50% OPC with GGBFS can reduce CO_2_ emissions by up to 0.5 t per m^3^ of concrete [64].

The production process involves rapid cooling of molten slag through powerful water jets, resulting in a fine-grained, glassy material composed of approximately 95% amorphous phase. Following drying and grinding, GGBFS typically achieves a specific surface area in the range of 4250–4700 cm^2^/g, exceeding that of OPC, and exhibits a specific gravity of approximately 2.85–2.90 g/cm^3^, slightly lower than that of cement (~3.15 g/cm^3^) [65,66].

Chemically, ground granulated blast-furnace slag (GGBFS) consists predominantly of CaO (30–50%), which governs its hydraulic activity; SiO_2_ (28–38%), responsible for pozzolanic reactivity; Al_2_O_3_ (8–24%) and MgO (1–18%), which influence binding reactions; as well as trace quantities of Fe_2_O_3_, SO_3_, Na_2_O, and K_2_O. The precise composition varies depending on the slag source, fluxes, and furnace feedstock.

Scanning electron microscopy (SEM) analyses reveal that GGBFS particles are angular, irregular in shape, and possess a rough texture with a relatively high surface area. This morphology increases the paste demand to ensure adequate particle coating, potentially affecting mix flowability. At the same time, the angular edges and rough surfaces improve interfacial bonding within the cementitious matrix, thereby enhancing density and reducing permeability. Hydraulic activity is strongly dependent on basicity, expressed as the CaO/SiO_2_ ratio, with higher values correlating with greater reactivity. The hydration of GGBFS is slower than that of ordinary Portland cement (OPC) and typically requires activation (e.g., through cement, lime, or alkalis). The main hydration product is calcium silicate hydrate (C–S–H), which contributes to strength development and durability. Conflicting reports on the optimum substitution level (30% vs. 40%) stem from differences in fineness, CaO/SiO_2_ ratio, and chemical activation. Finer, more reactive slags sustain higher dosages without early strength loss [67], whereas less reactive slags shift the optimum toward lower replacements. Akhlaq et al. [68] noted that balanced workability and strength typically occur in the 30–50% range, reinforcing the need for mechanistic evaluation of GGBFS in 3DCP rather than descriptive substitution ratios.

Partial replacement of OPC with GGBFS generally enhances workability up to substitution levels of approximately 40%, owing to the filler effect and improved particle packing. Beyond this threshold, workability tends to decline due to increased water demand and higher paste requirements; superplasticizers are therefore recommended. Studies consistently indicate that GGBFS replacement levels of 20–30% provide optimal improvements in compressive strength at both 28 days and later ages (56–90 days), driven by the combined effects of pozzolanic reactions and gradual hydration. From a 3D concrete printing perspective, the angular morphology and high surface area of GGBFS directly affect rheology at moderate replacements (~20–30%). They improve particle packing, filament cohesion, and extrudability, but at higher levels (>40%) they elevate yield stress and plastic viscosity beyond the printable window, impairing flow and continuity [43,69]. The slower hydration rate extends the open time, favoring extrusion, yet delays green strength development, which can compromise buildability. At higher replacement levels (60–80%), compressive strength typically decreases due to the dilution effect, i.e., a reduced fraction of reactive cementitious components. GGBFS was found to primarily contribute to mix cohesion, enhancing interlaminar stability and deformation resistance at higher exchange rates [47]. Furthermore, the incorporation of GGBFS in 3D-printed concrete introduces a pronounced anisotropic behavior, where mechanical properties strongly depend on the loading direction relative to the printing orientation. Shoueb [70] demonstrated that compressive strengths of printed specimens were consistently lower than those of conventionally cast counter-parts, with reductions ranging between 26–35% due to interlayer voids and lower density of the printed filaments. Directional testing further revealed that compressive strength in the X-direction was approximately 16% lower than in Y and Z, highlighting the weaker bonding across filament interfaces. In flexural performance, specimens tested in the Z-direction (perpendicular to layer planes) exhibited superior behavior: the modulus of rupture was more than 42% higher and the fracture energy absorption capacity about 55% greater compared to specimens tested in the Y-direction. This enhancement is attributed to the increased interfacial area and compaction achieved by successive layering in the vertical direction. Conversely, the Y-direction reflected the weakest interlayer bonding, limiting tensile and flexural resistance.

Rahmat et al. [69] reported that ~30% GGBFS achieves the most favorable balance between workability and strength, whereas higher replacement levels result in reduced flowability and increased porosity. Shreyas [71] found that ~40% GGBFS provides the highest mechanical strength and workability in conventional concrete, but for 3D printing applications, 30% substitution ensures superior production efficiency. The delayed hydration kinetics of GGBFS introduce a dual effect in 3DCP, extended open time supports pumping and continuous extrusion, yet the slower development of green strength compromises buildability and dimensional stability of early layers. Xu [43] observed that GGBFS addition extends the initial setting time from ~7.5 to ~9 min. At ~20% GGBFS, extrudability is significantly enhanced, with continuous filament lengths reaching 98 mm, reflecting improved flowability and homogeneity. Conversely, at 40% replacement, paste viscosity increases, filament length decreases, and print quality deteriorates. Buildability, defined as the ability of successive printed layers to retain shape without deformation, is maximized at ~20% GGBFS, whereas higher contents reduce geometric stability and dimensional accuracy.

Beyond mechanical and rheological aspects, GGBFS markedly improves durability. Its incorporation enhances resistance to acids, sulfates, carbonation, and chloride ion penetration. The filler effect also reduces capillary porosity, leading to lower water absorption, improved freeze–thaw resistance, and increased concrete density. In 3D printing applications, it functions as a rheology-modifying agent at optimal dosages, improving extrudability, shape stability, and interlayer bonding. Mixtures incorporating combined GGBFS and fly ash (0–40%) have demonstrated an optimal balance between strength and workability. At higher replacement levels, however, the use of chemical admixtures becomes necessary to ensure adequate viscosity and formability.

### 2.3. Addition of Coal Slag

Coal slag (CS) is produced under high-temperature coal combustion conditions, typically in boilers with forced air systems or in fluidized bed furnaces. It forms in the lower section of the combustion chamber, where it vitrifies as a result of rapid quenching with pressurized water. The material occurs as hard, glassy granules—often brick-red when fully combusted or dark gray when partially combusted—containing approximately 50% silica and significant amounts of alumina (Table 3) [72]. Depending on the cooling rate, coal slag may have a porous or dense structure. It exhibits a bulk density in the range of 700–1800 kg/m^3^, high water absorption of up to 20%, and porosity reaching 60%. The pronounced water absorption capacity of CS increases the static yield stress and accelerates structural build-up, thereby reducing the open time and adversely affecting pumpability. Furthermore, its inherent porosity intensifies internal friction, which results in elevated viscosity and diminished flowability through the nozzle [73]. Conversely, the rough and angular surface morphology enhances mechanical interlocking with the surrounding paste, thereby promoting stronger interlayer adhesion and improving the structural stability of the printed filaments [74]. Tao, Y. et al. demonstrated that, in 3D concrete printing, increased surface roughness and mechanical interlocking enhance interlayer bond strength and structural stability, with substrate roughness exerting a particularly significant effect on bond performance [75].

Its suitability for construction applications depends on limiting the presence of unburned carbon, sulfates, iron sulfides, and other contaminants that may adversely affect concrete performance [76]. Owing to its rough, irregular surface and favorable particle shape, CS is mainly used as a coarse aggregate in concrete, particularly in high-performance concrete (HPC), where it can improve paste–aggregate bond strength and reinforce the interfacial transition zone (ITZ). While its chemical properties are relatively stable, CS exhibits little pozzolanic reactivity; its role in concrete mixtures is primarily mechanical.

According to Smarzewski, the high porosity of CS promotes intensive absorption of mixing water, and both porosity and water absorption increase with higher CS content. In mixtures containing 30% CS, porosity and absorption increased by 11.3% compared to the reference mix, while density decreased by 7.8% [77]. A reduction in particle density decreases the self-weight of extruded mixtures, which can be advantageous for buildability. However, the concomitant increase in water uptake elevates the static yield stress and accelerates thixotropic structuration, thereby heightening the risk of pump or nozzle blockage at higher replacement levels. Consequently, the incorporation of superplasticizers or viscosity-modifying admixtures is commonly required to re-establish adequate flowability and to ensure sufficient open time for successful printing [78]. Regulatory standards define acceptable limits for loss on ignition, particle size distribution, foreign matter content, and natural radioactivity [79]. In addition to concrete applications, coal slag can also be used in geopolymers [80]. Moreover, in geopolymer systems, the reaction mechanisms and setting kinetics are strongly governed by the Si/Al ratio. Careful adjustment of CaO/Al_2_O_3_ and SiO_2_/Al_2_O_3_ proportions enables control over workability, early-age strength, and durability, which is particularly relevant for 3D printing applications [81]. Khargerdi et al. demonstrated that adding up to 10% CS by cement mass slightly increased compressive strength, reduced water absorption, and improved durability [82]. The authors’ previous studies have incorporated CS into foamed mixtures intended for 3D concrete printing. Results indicated that mixes containing CS combined with rigid fibers—such as basalt and glass fibers—achieved the highest flexural strengths of 4.14 MPa and 2.39 MPa, respectively [83]. Furthermore, partial replacement of cement with CS contributes to CO_2_ emission reductions. For concrete with CS, raw material production accounts for up to 90% of total emissions. At 30% CS content, the CO_2_ emission per unit of compressive strength (per MPa) was reduced by 0.13 kg compared to OPC concrete, making CS a favorable material from a sustainable construction perspective [84].

**Table 3:** Comparative analysis of coal slag oxide composition.

Oxides	Liu et al. [85]	Rezayt Khargerdi et al. [82]	Trinh et al. [80]	Rudziewicz et al. [83]	Ren et al. [84]
Na_2_O	0.58	1.21	0.43	-	1.78
MgO	2.06	1.65	1.40	1.20	1.47
Al_2_O_3_	22.19	22.84	24.2	31.94	15.93
SiO_2_	58.55	44.22	54.4	48.38	43.11
SO_3_	0.33	2.72	-	3.41	0.62
SrO	0.23	-	-	0.20	
K_2_O	2.36	4.5	3.59	2.61	1.30
CaO	6.28	1.28	1.30	1.73	16.69
TiO_2_	1.13	1.72	0.78	1.71	-
P_2_O_5_	0.22	0.40	0.08	0.60	-
Cr_2_O_3_	0.04	-	-	0.03	-
MnO	0.06	-	0.03	0.02	-
Fe_2_O_3_	5.66	19.5	-	7.66	18.60

Compressive strength increased substantially with the addition of CS—after 28 days, improvements ranged from 15.4% (10% CS) to 22.9% (30% CS) and after 730 days from 9.4% to 20.2%. Splitting tensile and flexural strengths also improved, with splitting tensile strength increasing by 8–9% at 28 days and 2–3% at 730 days. Flexural strength gains were particularly notable in early-age curing for mixtures with 10–20% CS [77].

### 2.4. Addition of Silica Fume

Silica fume (SF), a by-product generated primarily during metallurgical operations in electric arc furnaces, is extensively employed as a supplementary cementitious material in cement and concrete composites. Owing to its ultrafine particle size and high pozzolanic reactivity, SF significantly enhances both the mechanical performance and durability of cement-based materials. Its incorporation reduces matrix porosity, increases resistance to chemical degradation, and improves freeze–thaw durability [86].

From a sustainability perspective, the partial substitution of ordinary Portland cement with SF contributes to reducing CO_2_ emissions, thereby supporting the objectives of sustainable construction practices. The combined action of its microfiller effect and pozzolanic activity promotes refinement of the concrete microstructure, densification of the interfacial transition zone (ITZ), and an overall extension of service life under aggressive environmental conditions [87,88].

### 2.5. Waste Glass Powder

Ground waste glass (WGP) contains a high fraction of amorphous silica and, when sufficiently fine, exhibits pozzolanic reactivity beneficial to printable binders. Particle size is critical—the pozzolanic response becomes pronounced below ~300 µm. Fine fractions also provide a filler/packing effect, refining the microstructure and supporting strength development [62,89,90,91].

At low replacement levels (~5–15%) and after extended curing (e.g., 56–90 days), multiple studies report compressive-strength gains over reference mixes, attributed to the combined pozzolanic and filler mechanisms [90,91,92]. With respect to durability and microstructure, very fine glass particles (<75 µm) tend to mitigate alkali–silica reaction (ASR) and densify the ITZ by supplying nucleation sites for secondary C–S–H; by contrast, coarser or inert fractions can behave as flaws, weakening the ITZ and amplifying anisotropy [93,94,95,96]. Consistent with these mechanisms, reducing particle size and optimizing gradation at a fixed replacement level systematically improves performance; for example, at ~20% cement replacement, downsizing from ~74 µm to ~38 µm increased 14–90-day compressive strength, while tailored gradation improved interlayer adhesion and flexural strength, particularly under bending perpendicular to the print direction [97,98,99].

From a process standpoint in 3DCP, small–moderate WGP dosages improve matrix packing, can broaden the printability window, and stabilize buildability, whereas very high replacements may shorten open time unless viscosity is retuned [100].

Cross-reference: The use of WGP (as an SCM) is discussed in the summary of process-related effects and in the LCA context. In Section 2.6, recycled glass is treated solely as aggregate.

### 2.6. Addition of Construction and Demolition Waste

Recycled materials obtained from construction and demolition waste (CDW)—notably recycled coarse aggregate (RCA), recycled fine aggregate (RFA), crushed bricks, and waste glass—are increasingly recognized as sustainable alternatives to natural aggregates in concrete production. RCA, produced from crushed demolition concrete, is widely applied as a substitute for natural coarse aggregates, yielding notable environmental benefits by reducing landfill disposal and limiting the extraction of virgin resources. RFA, derived from the finer fractions of crushed concrete, can partially replace natural sand; however, its use is often associated with increased water absorption and reduced workability of fresh mixes. Crushed bricks, predominantly sourced from demolition debris, are also incorporated into concrete, primarily for non-structural applications due to their relatively lower strength and higher porosity compared with natural aggregates. Waste glass, when properly processed and sized, may serve as a fine aggregate in 3DCP. For binder-level use of ground waste glass (WGP) and particle-size effects, see Section 2.5 (“Waste Glass Powder”).

Beyond RCA, RFA, bricks, and glass, CDW encompasses a wide spectrum of additional materials with potential applications in concrete production, including wood, gypsum, plastics, metals, and asphalt. CDW can be broadly classified according to its origin, distinguishing between anthropogenic sources (e.g., construction, renovation, demolition activities) and natural disasters (e.g., earthquakes, floods, hurricanes, tsunamis). Although not all CDW fractions are suitable for direct incorporation as primary concrete constituents, several—once adequately processed—exhibit potential as supplementary fillers or additives. For instance, finely ground reclaimed asphalt pavement has been successfully incorporated into road and composite concretes, contributing to improved durability in specific applications. Gypsum waste, while often considered a contaminant in recycled concrete streams, has been investigated to a limited extent as a mineral additive. Plastics such as shredded PVC and polyethylene waste have been explored as lightweight aggregates or fibers to tailor rheological and mechanical properties, although challenges remain regarding long-term durability and fire resistance. Metal waste is typically recycled outside the concrete industry; in certain cases it has been employed as dispersed reinforcement or as a component contributing to improved thermal conductivity.

Beyond the need to ensure appropriate grading of waste-derived feedstocks, a major limitation is the risk of their inherent variability and potential contamination, and the resulting impact on mechanical properties; these factors necessitate rigorous mix-design strategies and strict quality-control measures. Sulfate-bearing gypsum residues, frequently present in fine recycled aggregates, may trigger internal sulfate attack and expansion; accordingly, standards cap water-soluble sulfate in aggregates (e.g., ≤0.2% by mass in EN 206 [101]), and best practice stresses selective demolition to avoid gypsum intrusion [102]. Adhered old mortar further elevates water absorption and porosity–often several-fold relative to natural aggregate—thereby shifting mix water demand and degrading the ITZ, which is consistently linked to strength penalties [103,104]. Impurities such as clay, gypsum/plaster, asphalt, wood, or paint have been reported to depress compressive strength even at modest contents, underscoring the need for upstream separation and contamination screening [105]. Moreover, limits on specific contaminants (e.g., bituminous/asphaltic constituents ≲ 10% by mass in EN 12620 guidance) should be combined with process controls such as moisture and absorption management (pre-saturation) and batch-wise rheology monitoring to maintain printable mixtures within the extrusion window [106,107].

Table 4 summarizes the mechanical properties of recycled aggregates used in three-dimensional concrete printing (3DCP). The incorporation of recycled sand, recycled coarse aggregate, waste glass, or plastics generally results in a reduction in compressive strength, particularly at higher substitution levels of natural aggregates [108,109]. This decrease in strength is primarily associated with the increased porosity of the matrix and the weaker bond at the aggregate–cement interface. For instance, the smooth surface texture of recycled glass particles may hinder effective bonding within the interfacial transition zone (ITZ) [110]. Furthermore, the presence of adhered residual mortar on recycled concrete aggregates can act as a structural weakness, negatively affecting both the mechanical integrity and long-term durability of the composite [111]. These microstructural features directly affect rheological parameters critical for extrusion. Increased porosity and adhered residual mortar elevate the static and dynamic yield stress as well as the plastic viscosity, which in turn shortens the open time and impairs pumpability and extrudability. For instance, Cavalcante et al. [112] reported that replacing 50% of cement with recycled concrete powder nearly tripled both static and dynamic yield stress compared with control mixes, moving the values outside the optimal extrusion range (280–600 Pa). These rheological shifts result in poor nozzle flow and reduced buildability unless compensated by superplasticizers or supplementary cementitious materials. Complementary studies [113] confirmed that partial replacement of Portland cement with recycled concrete powder (RCP) of up to 50% by mass produces similar trends in both rheological and mechanical behavior. Increases in static yield stress and thixotropic build-up were mainly attributed to physical effects associated with hydration kinetics and particle packing. Despite these rheological limitations, successful fabrication of a printed prototype with 50% cement replacement was achieved, demonstrating the technical feasibility of such high substitution levels. The deliberate adoption of this elevated ratio illustrates a more radical approach to reducing the environmental impact of printable mixtures driven by their high cement content while recognizing the inevitable trade-off in mechanical strength. Although the incorporation of these unconventional CDW-derived constituents remains largely experimental, their potential to support circular-economy strategies and reduce CO_2_ emissions positions them as a promising pathway toward more sustainable construction materials.

**Table 4:** Overview of Recycled Aggregate Behavior in 3DCP [114].

Recycled Materials	Replaced Materials	Replacement Ratio [%]	Particle Size [mm]	Fresh Properties	Mechanical Properties	References
Recycled sand	Natural sand	12.5–50	0.9	Increased extrudability	Slight reduction in compressive strength, limited effect on anisotropy	[109]
Recycled sand	Natural sand	50–100	3	Decreased workability	Increased porosity, reduced strength with higher replacement rates	[115]
Recycled concrete coarse aggregate	Quarry stone	50–100	5–12	Decreased flowability; increased buildability; shorter open time	Yield stress grows exponentially with time; shear modulus grows linearly -	[116]
Recycled concrete coarse aggregate	Nature coarse aggregate	50–100	5–12	Increased buildability	Decrease in strength with increased replacement ratio and age	[111]
Fine recycled aggregates (concrete & brick)	Natural fine aggregate	25–100	-	Fresh consistency ≥ 150 mm	Compressive strength (>50 MPa at 28 days); acceptable for 3D printing	[117]
Recycled brick aggregate	Natural aggregate	64	4.75	-	Reduction in strength due to porous RBA	[118]
Recycled glass	Natural sand	50	0.796	-	Increased porosity, negative impact on flexural strength	[99]
Recycled glass	River sand	100	0.15–1.7	Better flow properties	Lower buildability and mechanical properties	[110]
Recycled plastic eco-aggregate (RESIN8)	Natural sand	5–15%	<1 mm/<5 mm	Higher flowability, lower thixotropy; poorer buildability	Decrease in compression and flexural strength; improved interlayer bond strength	[119]
Polyethylene terephthalate (PET)	Natural sand	10–50	4	Decreased buildability	Decreased buildability and mechanical properties, improved freeze–thaw resistance	[120]
Recycled plastic waste	Natural sand	5–15	5	Increased flowability and porosity	Decreased strength	[119]
Recycled crumb rubber	River sand	15	2.36	-	Anisotropic compressive strength	[121]
Waste tire rubber	Natural sand	25–100%	1–3	Decreased workability	-	[122]
Waste tire rubber	Natural sand	50	1–3	-	Lower environmental impact and energy effectiveness in 3D printing	[123]
Rubber aggregates	Natural sand	5–15	0.1–3	Decreased extrudability, improved buildability	-	[124]
Copper slag as fine aggregate	River sand	up to 100%	-	Increased workability	Mechanical properties can be maintained at full sand replacement (requires mix optimization)	[125]
Steel slag as fine aggregate	River sand	up to 100%	-	Mixtures fall within the printability window; good buildability	Printable elements; strength depends on steel slag aggregate-to-cement ratio (SA/C)	[126]

Recycled Concrete Aggregate (RCA) is a secondary raw material obtained through the processing of construction and demolition waste, primarily originating from dismantled concrete structures. By definition, RCA consists of natural aggregate particles partially coated with remnants of hardened cement paste (adhered mortar), which strongly influences its physical, mechanical, and durability-related properties. The production process typically involves selective demolition, mechanical crushing—most often performed in two stages—and subsequent separation of contaminants such as reinforcing steel, wood, or plastics [127]. The processed aggregate is subsequently classified into coarse fractions (5–32 mm) and fine fractions (<5 mm), the latter referred to as Recycled Fine Aggregate (RFA), which is particularly relevant for 3D concrete printing (3DCP) due to pumping and nozzle size constraints in printing systems [73,128]. The incorporation of fine recycled concrete aggregates (FRCAs, <4 mm) into 3D-printable mortars has been shown to increase static yield stress and plastic viscosity owing to their elevated surface area, porosity, and residual adhered mortar. At a 50% replacement level, Sbardelotto et al. [129] observed reduced flowability and shortened open time, accompanied by enhanced structural build-up and interlayer stability. Consequently, FRCA can improve buildability but adversely affect extrudability, necessitating higher dosages of superplasticizers to maintain adequate nozzle flow.

#### Impact of RCA/FRCA on Extrusion-Relevant Rheology: Quantitative Benchmarks

For extrusion systems in 3DCP, it is useful to consider three operating “windows” of a printable mixture: pumpability (low resistance to flow in the line), extrudability (governed mainly by dynamic yield stress), and buildability (governed mainly by static yield stress). Consolidated reviews and experimental studies report typical ranges: a pumpability zone on the order of tens of Pa in the line (higher values promote blockages); an extrudability window often ~0.1–0.5 kPa for dynamic yield stress (occasionally up to ~0.9 kPa); and buildability requiring static yield stress ≈ 500–600 Pa (with plastic viscosity ~2–10 Pa·s, ~8 Pa·s in representative studies) for stable layer-by-layer deposition; higher viscosities and yield stress improve green load-bearing capacity but shorten open time and increase required pump pressures [130,131].

Data for mixtures containing RCA/FRCA show a systematic increase in static and dynamic yield stress and in plastic viscosity, as well as an accelerated structural build-up, which improves buildability at the expense of extrudability and open time. For 20% RCA, reports indicate dynamic yield stress ≈ 944.5 Pa (t = 0 min) rising to ≈ 1676.4 Pa (t = 30 min)—outside the typical nozzle range for extrusion; these values were reduced using a superplasticizer without increasing w/b (this also holds for FRCA) [123,124]. Introducing 25% and 50% FRCA increased static/dynamic yield stress and the rate of structural build-up; in studies on mortars with recycled sand, buildability increased by ~33% and ~83%, respectively, while flowability decreased and open time shortened. Review papers emphasize that keeping FRCA-bearing mixes within the ~0.1–0.5 kPa (dynamic) range, occasionally up to ~0.9 kPa depending on system: superplasticizers (often with tailored polymer architecture), which lower yield stress and plastic viscosity at constant w/b; SCMs/particle packing, to counter the effects of porosity and specific surface area of adhered mortar; and optionally rheology regulators (e.g., sodium gluconate) to slow the growth of yield stress over time and extend open time. As operational targets for FRCA mixtures, we recommend: τ_0_,dyn (t = 0) ≤ 600 Pa, the time-dependent increase in dynamic yield stress during printing kept within the capacity of the pump/system, and plastic viscosity maintained at the lower end compatible with the required cross-section stability of the filament [130,131,132,133,134,135].

RCA particles differ from natural aggregates (NAs) primarily in terms of bulk density, which typically ranges between 2.1–2.4 g/cm^3^, compared to 2.5–2.7 g/cm^3^ for NAs, a reduction of up to 17% [136]. Nevertheless, their rougher surface morphology may improve mechanical interlocking with fresh paste, potentially benefiting interlayer bond strength in 3D-printed structures. Beyond the well-recognized weakening of the interfacial transition zone (ITZ) due to adhered mortar or smooth glassy surfaces, the pore structure of recycled aggregates also governs interlayer adhesion in 3DCP. Liu et al. [94] reported that larger and irregular pores concentrated near the aggregate surface disrupt paste continuity across layers, reducing local bonding and promoting microcracking at the interface. This highlights that porosity is not only detrimental to bulk strength but also a decisive factor in anisotropic behavior of 3D-printed elements, where weak interfaces become the primary failure planes.

In response to these challenges, research efforts have increasingly shifted towards RFA in 3DCP applications. Compressive strengths reported for RFA-based mixtures range from 25 to 60 MPa [137,138]. Reddy et al. [32] demonstrated that the compressive strength of 3D-printable concretes varies significantly with the level of RFA replacement. Similarly, Khatib [139] reported that partial replacement of natural fine aggregate with RFA in the range of 25–75% yields compressive strengths comparable to control mixes, whereas complete replacement results in notable strength reduction. Strength losses associated with increasing RFA contents have been observed consistently across all build directions (X, Y, Z), with reductions ranging from 9.4–29.2% in X, 12.5–33.1% in Y, and 14.9–40.0% in Z [140,141].

In addition to conventional RFA, other secondary materials with appropriate particle size distributions have been investigated for 3DCP, including crushed waste brick and recycled glass. Brick waste, predominantly derived from the demolition of masonry and ceramic elements, typically ends up in landfills, contributing to land-use pressure and environmental degradation [142]. Consequently, its reuse in 3D-printed concrete (3DPC) has attracted considerable interest [143]. When crushed to fine fractions (<4 mm), waste brick can function as RFA, while ceramic brick powder (CBP), obtained from finely ground waste brick, has potential as a partial cementitious binder replacement.

Due to its porous structure, brick aggregate exhibits higher water absorption and lower density relative to natural aggregates, which can adversely affect mechanical properties. Nonetheless, brick aggregate demonstrates strong adhesive characteristics and favorable thermal resistance. Experimental findings suggest that higher porosity may improve high-temperature performance by facilitating vapor release and reducing internal stresses; however, deterioration of interlayer bonding is reported at 600–800 °C [144]. At smaller particle sizes (<2 mm), brick waste has in some cases improved compressive strength compared to conventional concrete, by as much as 8.24% (28.90 MPa vs. 26.70 MPa), while splitting tensile and flexural strengths were maintained or slightly enhanced up to 20% replacement, with maximum flexural strength reaching 6.97 MPa. Aboalella et al. reported that replacing 50% of fine aggregate with crushed brick resulted in compressive strength increases of 23% at 7 days, 28% at 28 days, and 19% at 56 days. The optimal performance was observed when 50% of coarse aggregate was replaced with RCA and 50% of fine aggregate with crushed brick, yielding compressive, tensile, and flexural strength increases of 32%, 28%, and 26%, respectively [145]. Conversely, Zheng et al. demonstrated that complete replacement of natural aggregate with brick aggregate reduced compressive strength by 11–12% at w/c = 0.55 and 13–17% at w/c = 0.35 after 28 and 56 days [146]. Their earlier work confirmed that brick aggregate mixtures exhibited high printability and repeatability, attributed to optimized particle grading and porosity, which improve structural stability during extrusion and ensure uniform flow [147]. Other studies confirmed that printed elements incorporating brick aggregates displayed satisfactory surface finish and dimensional accuracy, including the successful printing of a 390 mm-high façade element. However, partial substitution levels remain critical: replacing 64% of natural aggregate with brick aggregate reduced cube compressive strength by 25% (39.9 MPa vs. 53.1 MPa), interlayer tensile strength by 20% (1.37 MPa vs. 1.72 MPa), and compressive strength in build directions D1 and D3 by 14% and 20%, respectively [118].

Ground brick powder (CBP) has been shown to exert a non-linear influence on compressive strength: performance increases at replacement levels up to 10%, decreases at intermediate levels, and slightly increases again at higher dosages, with 10% identified as the optimum. In 3DPC, incorporation of 10% CBP, combined with appropriate mix adjustments, enhanced workability, compressive strength, and reduced structural anisotropy [148]. Both crushed brick and CBP therefore represent promising options for 3DCP, offering favorable rheology, good printability, and partial replacement of conventional raw materials. Their use modifies mechanical performance, but, with optimized mix proportions, durable and aesthetically appealing printed elements can be achieved while reducing environmental impact. In the study [95] it was observed that appropriate processing (e.g., particle gradation) of CBP combined with RFA results in a more homogeneous particle distribution and improved crack control after exposure to elevated temperatures. This behavior directly influences printing stability and mechanical performance. In this context, the interfacial transition zone (ITZ) has been described as a privileged site for crack initiation, owing to its higher local water-to-cement ratio and weaker microstructure compared to bulk paste [149]. When coarser, porous, or chemically inert fractions are used, such as insufficiently reactive CBP or oversized glass particles, they tend to behave as flaws within the ITZ, undermining cohesion and amplifying anisotropy under load. Conversely, finely ground and reactive fractions densify the ITZ through additional C–S–H formation, thereby improving interlayer bonding in 3D-printed concretes.

Similarly, recycled glass, when crushed to particle sizes comparable to natural sand (<4 mm, often <0.3 mm), can serve as RFA. Binder-level effects of WGP are covered in Section 2.5; here, recycled glass is discussed only as aggregate.

Ting et al. examined the rheological and mechanical performance of 3D-printed cementitious materials incorporating recycled glass [110]. In their study, river sand was fully replaced with a blend of glass particles, consisting of ~25% fine-to-medium fractions (500–1700 µm) and ~75% very fine fractions (150–710 µm). Their findings indicated that glass addition facilitated the pumping and delivery of fresh mortar through the printing nozzle. Flexural strength of glass-modified mixes followed trends similar to compressive strength. Kim et al. investigated the replacement of fine aggregate with recycled glass at levels ranging from 50% to 100% by volume. With a water-to-binder ratio of 0.45, flexural strength decreased by 9% and 14% compared to the reference mix [150]. Conversely, other studies reported flexural strength improvements of 25–33% when the bending direction was perpendicular to the printing orientation, although decreases of 8–20% were observed when tested parallel to the printing layers [99]. Likewise, Jiao et al. reported flexural strength increases of 2%, 1%, 5%, and 1% at recycled glass replacement levels of 25%, 50%, 75%, and 100%, respectively [151]. Previous studies have demonstrated that the flexural strength of printed layers varies with printing orientation, thereby confirming the critical role of interlayer adhesion and microstructural integrity [152].

In contrast, Ting et al. found that compressive strength of glass-containing concrete was approximately 50% lower than that of conventional sand-based concrete (16 MPa vs. 33 MPa), while flexural strength decreased by 30% (21 MPa vs. vs. 30 MPa) [110]. These differences can largely be attributed to the weaker bond between recycled glass particles and the cement matrix compared to that observed with river sand concrete [135,153].

Table 5 consolidates material-specific implications for 3D concrete printing (3DCP), focusing strictly on process-relevant advantages and disadvantages (pumping, extrusion, printability window, buildability, interlayer bonding).

### 2.7. Long-Term Durability of 3D-Printed Concrete Mixes with Recycled Materials

In 3DCP, durability is governed not only by mix composition but also by anisotropy and the quality of interlayer interfaces, which may act as preferential pathways for moisture and ionic transport; the Y-direction is often the most vulnerable in terms of interlayer cohesion, directly affecting freeze–thaw (F–T) resistance and chloride ingress [114]. Recycled fine/coarse aggregates (RFAs/RCAs) tend to raise water absorption and matrix discontinuity, while polymer recyclates (e.g., PETs) alter stiffness and fracture behaviour—both effects amplifying the role of interfacial regions [109,115,116,117,120,121]. This aligns with a recent review highlighting interlayer quality and mechanical anisotropy as key to structural performance, alongside a synthesis of 3DPC environmental impacts [163].

Ground granulated blast-furnace slag (GGBFS) densifies the microstructure (filler action and additional C–S–H), lowers capillary porosity, and typically enhances both F–T performance and resistance to chloride transport; in 3DCP, a practical dosage window is ~20–30% to balance tightness with buildability and the printing time window [69,71,73]. Silica fume (SF) further seals the ITZ and improves F–T—particularly valuable for mixes with elevated absorption (e.g., with RFA) [86,87,88]. Fly ash (FA) contributes pozzolanically and, in hybrid systems with GGBFS, reduces chloride migration/diffusion while helping to retain printability [154,155,156]. PET can improve F–T at moderate contents, though often at the expense of strength/buildability (Table 6) [120].

Recommended practice is to minimise open porosity through GGBFS (~20–30%) plus SF (~5–10%)—optionally in hybrids with FA—together with particle packing and superplasticizers (SPs) instead of increasing w/b. Control of viscosity, the build-up of yield stress (τ_0_), and the open time is essential to limit microgaps at interfaces and maintain interlayer continuity, particularly at higher RFA/RCA replacements. Verification should report durability with directional resolution: F–T after 56/100 cycles (mass loss and relative reductions in E and fct/fc) and rapid chloride migration/diffusion tests—both distinguished by orientation with respect to layers (X/Y/Z). Complementary measurements include water absorption, sorptivity, open porosity and an anisotropy map [69,73,87,88,129,130,132,154,155].

**Table 6:** Effect of selected constituents on F–T and chloride transport.

Recycled Constituent	Typical Effect on F–T	Typical Effect on Chlorides	3DCP Process Levers	Reference
GGBFS (20–30%)	↑ F–T (densification, ↓ porosity)	↓ migration/diffusion (pore refinement)	Balance against open time; >40% often needs SP	[69,71,73]
Silica fume (5–10%)	↑ F–T; sealed ITZ	↓ porosity → reduced ingress	Tight viscosity/extrusion control; w/b and SP	[86,87,88]
Fly ash (FA)	neutral to ↑ F–T (indirect via densification)	↓ migration/diffusion (pozzolanic; in GGBFS hybrids)	GGBFS + FA hybrids; manage rheology and open time	[154,155,156]
PET/other plastics	sometimes ↑ F–T at moderate contents	no single trend (mix-dependent)	Limit dosage; pair with SCMs (GGBFS/SF/FA)	[120]
RFA/RCA (25–50%)	risk of ↓ F–T (↑ absorption, weaker interfaces)	↑ vulnerability to ingress (interlayer pathways)	SP, particle packing, curing; control τ_0_ and print window	[94,109,115,116,117,133,134,135]

The literature-supported route to mitigating F–T damage and chloride ingress in recycled 3DCP mixes is a low-porosity matrix built around GGBFS + SF (with optional FA hybrids) and high-quality interlayer bonding. Key gaps remain: sparse F–T and chloride data at high RFA/RCP contents, systematic directional durability testing (Y vs. Z), and stronger links between τ_0_ kinetics and transport at interlayer interfaces.

## 3. Calculation of the Benefits of Using Secondary Raw Materials

The environmental aspect of incorporating recycled raw materials into concrete mixtures, both for conventional construction and 3DCP technology, is of paramount importance. To properly balance the mix composition while accounting for ecological impact, appropriate standards and comparative parameters are required. The carbon footprint is a measure of the total greenhouse gas emissions associated with a product or process, expressed as carbon dioxide equivalents (kg CO_2_-eq). In practice, it corresponds to the GWP100 indicator (Global Warming Potential over a 100-year horizon), which aggregates the impact of CO_2_, CH_4_, N_2_O, and other gases into a single metric.

For construction products—including concrete—the carbon footprint is calculated within the framework of life cycle assessment (LCA), a structured methodology for assessing environmental impacts “from cradle to grave” or “from cradle to gate” [164,165,166].

LCA consists of four main steps: (1) goal and scope definition (system boundaries), (2) life cycle inventory (LCI—compilation of all inputs/outputs: raw materials, energy, emissions), (3) life cycle impact assessment (LCIA—conversion of flows into indicators such as GWP), and (4) interpretation of results. Critical elements here are the system boundaries and the functional or declared unit. In the case of concrete mixtures, the practical reference unit is typically 1 m^3^ of concrete with defined mechanical properties.

In the construction sector, life cycle modeling is conventionally divided into modules A–D [164,167,168]:

- A1–A3 (product stage): raw material acquisition, binder and admixture production, mixture manufacturing (the so-called cradle-to-gate).
- A4–A5 (construction): transportation to site and placement.
- B (use stage): service life of the element/structure (in concrete, carbonation is particularly relevant—gradual CO_2_ uptake by the material).
- C (end-of-life): demolition, crushing, recovery, or landfilling.
- D (beyond system boundaries): credits/burdens from substitution of primary materials due to recycling or energy recovery.

Modern reporting frameworks for construction products distinguish between carbon footprint components, e.g., GWP-fossil (from fossil fuels), GWP-biogenic (from biogenic carbon), GWP-LULUC (from land-use change), and GWP-total (sum). For comparing mixtures, results are most often expressed as GWP-total (kg CO_2_-eq/m^3^) for the A1–A3 stage, and—where possible—the full A1–A3, C, D profile, depending on the study’s scope [164,169].

In practice, the dominant emission sources in concrete mixtures are:

- Production of cementitious binders, particularly process emissions from clinker decarbonation (calcination) and kiln fuel/energy use;
- Transportation of raw materials and fresh concrete;
- Manufacturing (electricity and heat at batching plants);
- End-of-life stage (crushing, sorting, transport, disposal).

Therefore, low-carbon mix design typically relies on: (i) reducing clinker content through SCM substitution, (ii) replacing natural aggregates with recycled fractions, (iii) optimizing logistics and energy use, and (iv) accounting for recycling credits (D) and carbonation where methodologically justified. Complementing these standards, a 2024/2025 review integrates mechanical performance data of 3D-printed concretes with life-cycle assessment findings, linking mix-design choices to environmental outcomes [163].

The methodology for calculating the carbon footprint and related indicators is standardized mainly through:

- ISO 14040/14044—general principles and requirements for LCA (overall framework) [167,168];
- ISO 21930:2017—rules for Environmental Product Declarations (EPDs) of construction products (complementary to ISO 14025) [169];
- EN 15804:2012+A2:2019—European core PCR for all construction products: mandatory modules A1–A3, C1–C4, D; expanded set of indicators, including the breakdown of GWP into fossil, biogenic, LULUC, and total [164].

Based on these standards, various tools have been developed to support environmental performance calculations. Among the most widely used is SimaPro [170], a comprehensive LCA software with extensive databases (e.g., ecoinvent, Agrifootprint). An open-source alternative is openLCA [171], also compatible with ecoinvent. In the construction sector, tools such as eToolLCD [172] or Tally (Revit plugin) [173] are frequently applied, as they can be integrated with BIM. In addition, One Click LCA [174] offers both standard LCA functionalities and an EPD generator for concrete, while the GCCA Industry EPD Tool [175] provides a sector-specific calculator for aggregates, clinker, cement, concrete, and precast elements.

For recycled concrete mixtures (e.g., with recycled aggregate, RCA or clinker substitutes, SCM such as blast furnace slag or fly ash), four modeling decisions are particularly important [164,169,176,177]:

- Allocation and cut-off criteria: whether and how environmental burdens are assigned to secondary raw materials entering the system (commonly counted from the “end-of-waste” point).
- Carbonation: inclusion of CO_2_ uptake during service life (B1) and after crushing at end-of-life (C3); critical for comparing scenarios.
- Module D: accounting for substitution benefits (e.g., recycled aggregate replacing natural aggregate in subsequent cycles).
- Data quality and representativeness: selecting appropriate datasets (e.g., regional electricity mixes, cement production parameters, transport processes) consistent with relevant PCR/EPD rules.

In the literature, the carbon footprint of concrete is usually reported as GWP100 per 1 m^3^, most commonly for the A1–A3 stage (“cradle-to-gate”), while more recent studies also include carbonation and module D in line with EN 15804+A2/EN 16757. Reported values for “reference” mixtures vary widely, e.g., ~348 kg CO_2_-eq/m^3^ in production studies, ~323–332 kg CO_2_-eq/m^3^ for “cement-only” mixes with natural aggregates, or reductions from ~450 to ~250 kg with aggressive clinker substitution by SCM—clearly demonstrating the critical role of clinker content and system boundaries [165,166]. Table 7 presents intervention-specific LCA results reported across different studies, Table 8 compiles representative GWP values for conventional and blended concrete mix designs, and Table 9 specifically addresses waste-derived constituents in 3D-printed concrete. Collectively, these tables provide a structured synthesis of current evidence, progressing from general assessments to 3DCP-specific contexts.

For recycled aggregate concrete (RAC), the picture is less consistent: replacing natural aggregate alone rarely achieves reductions comparable to SCM substitution, but it improves A1 emissions (lower aggregate production footprint) and—in end-of-life scenarios—increases CO_2_ uptake through carbonation, which is increasingly accounted for in B1/C3 and D in recent EPDs. Additionally, CO_2_ curing technologies can further reduce “cradle-to-gate” emissions by several to over ten percent, although literature emphasizes the sensitivity of results to SCM/aggregate transport and uncertainties in carbonation modeling [178,179,180,181].

**Table 7:** Summary of studies (mixtures, methods, and GWP results).

Intervention/Mixture	Scope & Method	Tool/Database	Result/Change	Reference
“Reference” concrete (production)	A1–A3, IPCC	–	~348 kg CO_2_-eq/m^3^	[165]
NAC “cement-only” vs. +25% FA (SCM)	A1–A3, IPCC 2013	openLCA; ecoinvent	NAC 323–332; −8–17% at 25% FA	[166]
“Low-clinker” mixes (high SCM)	A1–A3 (various LCAs)	various tools	~450 → ~250 kg with high SCM	[182]
Prefabricate, CO_2_-curing; NA/RCA/MCA	A1–A3 (incl. bound CO_2_ balance)	(measurement + inventory)	221.26 (NA), 204.38 (RCA), 210.05 (MCA)	[179]
RAC, accounting for carbonation in life cycle	B1/C3 (uptake)	–	4.9–16.4 kg CO_2_/m^3^ absorbed (30–100% RCA; 50 years)	[178]
Workflow for carbonation (EN 16757)	B1 (k-values)	digital model	Rate ~1.59 mm·year^−0^·^5^ (per EN 16,757 for external exposure)	[183]
Effect of FA/slag transport	A1–A3	–	Higher FA/slag content reduces GWP, but long transport distances may offset gains	[180]
CO_2_-cured blocks (comparative)	A1–A3 (variants)	–	292–454 kg/m^3^ (scenario-dependent)	[184]
Recycled aggregate production	A1 (aggregates)	–	–70.7% impact vs. natural aggregate	[185]

**Table 8:** Comparative carbon footprint of different concrete mix designs (GWP100, cradle-to-gate, per 1 m^3^).

Concrete Type/Mix Design	Main Features	Typical GWP100 [kg CO_2_-eq/m^3^]	Key Factors Affecting Results	References
Conventional concrete (OPC only)	100% clinker cement, natural aggregates	~320–450	High process emissions from clinker calcination; energy-intensive	[186,187,188,189]
Blended concrete with SCM	20–50% clinker replaced by FA, GGBFS, SF, MK	~250–330	Reduction depends on SCM level and transport distance	[188,190,191]
Recycled aggregate concrete (RAC)	RCA/RFA replacing natural aggregates	~300–420	Lower A1 emissions; carbonation in B1/C3; less impact vs. SCM	[192,193,194]
Low-carbon hybrid mixes	SCM + RCA combined	~220–300	Synergistic effect; possibility of CO_2_ curing for further reduction	[193,194]
Geopolymer concrete	Alkali-activated binders from industrial by-products	~150–250	Very low process emissions; strongly influenced by activator type and regional energy mix	[195,196,197]

**Table 9:** Carbon footprint reduction achieved by using waste-derived raw materials in mixes for 3D concrete printing (3DCP), as reported in the literature.

Recycled Material Used in 3DCP Mix	Modification/Role in the Mix	LCA Type (Scope)	GWP Effect (vs. the Reference Mix in the Study)	Key Notes	Reference
Fly ash (FA)	Partial replacement of binder in printable mixes (SCM/precursor in geopolymers)	Mix-level LCA for 3D-printed building; cradle-to-gate for materials	Higher GWP than GGBFS within the same study set: FA ≈ +37% vs. GGBFS; equivalently ~393 kg CO_2_e/m^3^ if GGBFS = 287 kg CO_2_e/m^3^	Comparison is across 3DCP material variants; the lowest footprint in that set was GGBFS; FA and WGP were higher	[198]
Ground granulated blast-furnace slag (GGBFS)	Partial replacement of cement in printable mixtures	Mix-level LCA (cradle-to-gate) per unit volume	Lowest GWP in the assessed set: ~287 kg CO_2_e/m^3^; in the same analysis FA ~37% higher, WGP ~50% higher	Authors recommend GGBFS as the most effective low-carbon option among compared by-products for 3DCP	[198]
Coal slag (CS) (boiler slag)	Potential SCM/fine fraction in 3DCP	data gap for mix-level 3DCP LCA	–	Material reports exist, but no standardized cradle-to-gate GWP for printable mixes	–
Silica fume (SF)	Low-dosage SCM in printable mortars	data gap for mix-level 3DCP LCA	–	Directionally reduces clinker content, but mix-level 3DCP LCA with explicit GWP is missing	[199]
Recycled concrete powder (RCP/RCBP)	Up to 50% binder replacement in printable paste/mortar	Mix-level LCA (cradle-to-gate); FU = 1 m^3^ paste/mortar	Up to ~−62% CO_2_e vs. OPC reference (while maintaining printability after rheology tuning)	Strong sensitivity to yield stress and printability window; admixture adjustments required at high RCP contents	[112,113]
Fine recycled aggregate (fRA) (from 3DPC/CDW)	Replacement of sand with concomitant binder reduction	Mix-level LCA for 3DCP (cradle-to-gate)	Up to ~−48% CO_2_e at the highest fRA levels with cement reduction; ~−20% feasible at ~20% *v*/*v* without major performance loss	Effect largely driven by clinker reduction; at >20% *v*/*v* monitor green strength and printability window	[159]
Ground waste tire rubber (GWTR)	Partial replacement of sand; printable mixes compared with casting	Mix-level LCA (cradle-to-gate)	Environmental credit for GWTR (negative GWP for the aggregate module under “avoided burden”); additionally ~−9.5% GWP when printing vs. casting the same mix	Total reduction depends on allocation for GWTR and rubber content; cement remains the dominant GWP contributor	[123]
Waste glass powder (WGP)	10–30% OPC replacement (SCM) in printable mortars	data gap for mix-level 3DCP LCA	–	Directionally beneficial via clinker reduction; some studies rank GGBFS < FA < WGP in GWP (relative SCM ranking)	[100]

In summary, the assessment of the carbon footprint of concrete mixtures with recycled materials requires a consistent life cycle perspective, clear methodological assumptions, and reliable datasets. The main levers for emission reduction remain clinker substitution with SCMs and, to a lesser extent, aggregate recycling, both complemented by optimized logistics and accounting for carbonation and end-of-life credits. Advanced LCA software and standardized frameworks such as ISO 14040/44 and EN 15804 provide robust tools for benchmarking environmental performance. As research shows, properly designed recycled concrete mixtures can significantly reduce GWP while maintaining structural integrity, confirming their role as a key pathway toward sustainable and decarbonized construction.

## 4. Material Reuse in 3D-Printed Concrete: A Comparison with Traditional Methods

**Figure 4:**
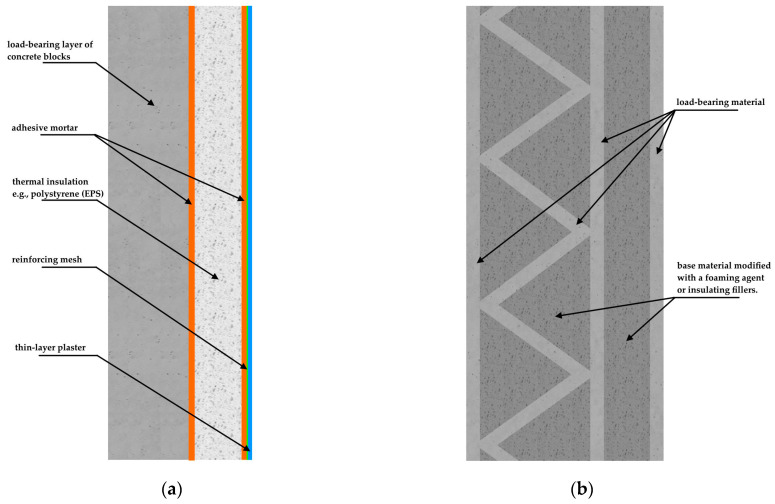
Schematic cross-sections of insulated walls: (**a**) traditional multi-layer masonry (concrete blocks with ETICS using EPS); (**b**) 3D concrete printing (3DCP) wall with load-bearing material and base material modified with a foaming agent or insulating fillers.

In the context of construction and demolition waste (CDW), 3D-printed concrete (3DCP) offers a comparatively cleaner and more traceable waste stream than conventional multilayer wall systems. By design, many 3DCP envelopes minimize or eliminate auxiliary layers that complicate end-of-life processing—such as polymeric thermal insulation, gypsum plasters, adhesives, glass/aramid meshes, or complex façade laminates—thereby reducing contamination and facilitating selective demolition (Figure 4). The digital nature of production (known mix designs, documented print paths, and layer counts) further improves material provenance and homogeneity, which are decisive for high-quality secondary aggregates and for closed-loop strategies (e.g., re-introducing recycled concrete aggregate, RCA, into new 3DCP mixes).

Practical advantages of 3DCP waste over conventional CDW include:

- Lower contaminant load—fewer gypsum, wood, plastics, and adhesive residues that otherwise depress concrete strength when recycled [200];
- Simplified disassembly and shorter processing chains—often fewer steps before crushing/screening, with reduced handling, storage, and time;
- Reduced metallic content—many 3DCP systems rely on geometry and localized reinforcement, limiting extensive steel meshes and hazardous resin-bonded mortars common in ETICS or render systems;
- Logistics benefits—less bulky polymeric insulation (e.g., EPS) to transport and compact; EPS’s very low bulk density makes long-haul transport particularly inefficient in conventional retrofits and demolitions;
- Higher traceability—digital fabrication enables accurate mass balances and targeted reuse pathways;
- Compatibility with closed-loop reuse—on-site or local crushing/sieving can yield RCA tailored to new 3DCP mixes, limiting primary aggregate demand.

Figure 4 contrasts two representative insulated wall assemblies: (a) a traditional multi-layer masonry wall comprising a load-bearing course of concrete blocks with an external thermal insulation composite system (ETICS) based on expanded polystyrene (EPS), adhesive mortar, reinforcing mesh, and thin-layer plaster and (b) a 3D concrete printing (3DCP) wall composed of printed load-bearing ribs and a base cementitious material modified with a foaming agent or insulating fillers. This comparison highlights the stark end-of-life contrast between conventional systems and 3DCP. In conventional walls, effective recycling hinges on strict source separation: reinforcement meshes embedded in adhesive mortars are often treated as hazardous and require specialized processing; plastics are energy-intensive to produce and difficult to recover economically; laminated or coated architectural glass, although technically recyclable, remains energy-demanding and materially complex. Moreover, popular thermal insulants pose practical barriers: EPS produces large volumes of low-density waste (transport-intensive), while mineral wool—though recyclable in principle—depends on costly, infrastructure-limited routes and currently low demolition volumes (mainly 1970s–1980s stock), which undermines profitability. Meeting selective demolition and waste-purity requirements remains essential to avoid down-cycling in both cases, but it is intrinsically easier for the streamlined 3DCP assembly [200,201,202].

**Figure 5:**
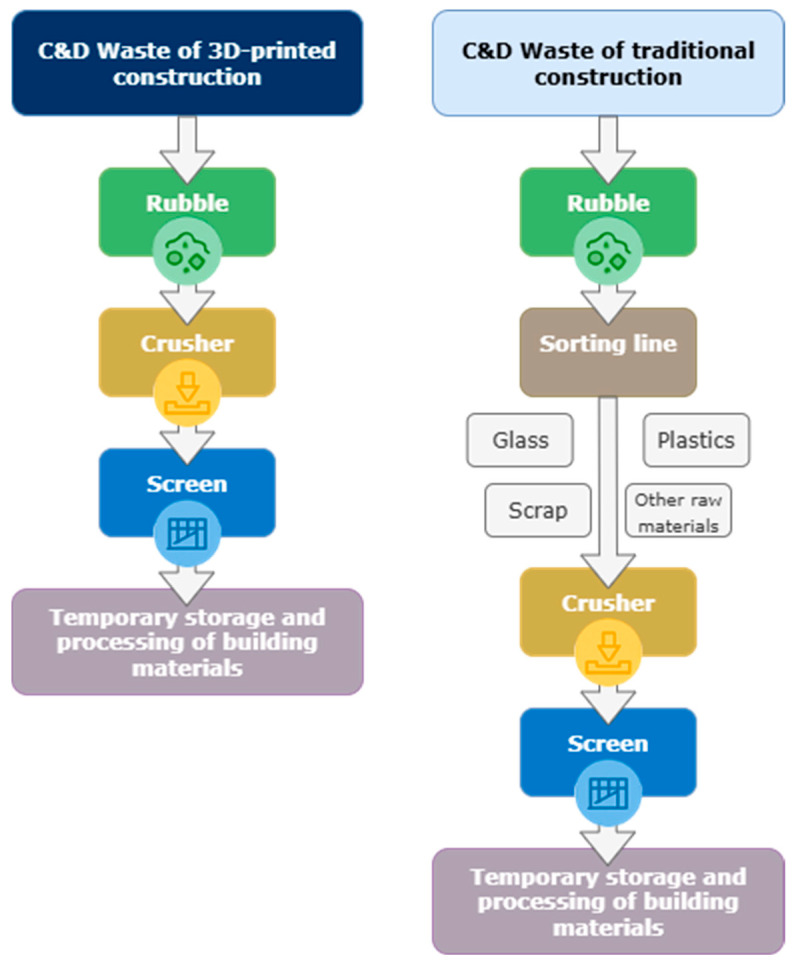
Recycling chain of CDW of 3D-printed and traditional construction.

By contrast, 3DCP can reduce the number of processing stages from demolition to usable RCA—often bypassing the removal of adhesive layers, mesh detachment, or composite façade separation. This shortens the recycling chain (Figure 5), lowers equipment hours and temporary storage needs, and can improve RCA quality by limiting fines contamination. Figure 5 illustrates that the recycling chain of 3DCP waste is generally shorter and less complex compared to conventional demolition materials. Due to the absence of sorting processes for plastics, glass, or composite residues, 3DCP rubble can be directed straight to crushing and screening, thereby minimizing handling steps and reducing potential material losses. From an LCA perspective, this results in lower energy demand, reduced requirements for transport and storage, and decreased CO_2_ emissions associated with processing. Consequently, 3DCP assemblies not only streamline logistical operations but also enable higher-quality RCA recovery and foster more resilient closed-loop recycling strategies. As highlighted by experimental evidence, even modest levels of contaminants (e.g., lime plaster—7% vol., soil—5%, wood—4%, gypsum—3%, asphalt—2%, vinyl-acetate paints—0.2%) may reduce compressive strength by up to ~15% [200]. Mitigating such inputs is inherently easier with the streamlined assemblies typical of 3DCP. In addition, 3DCP can offset upstream impacts through reduced material intensity and shorter execution times, decreasing vehicle movements and site energy demand. The primary structural material—concrete—remains almost fully recyclable into secondary aggregates, enabling loop-closing strategies whereby demolition waste from printed buildings is reprocessed and redeployed in subsequent 3DCP projects. Advances in sustainable construction are not limited to material substitution but also involve predictive optimization of engineering processes. Studies on tunneling thrust prediction using multi-channel data fusion [203] illustrate how such computational techniques can reduce inefficiencies, a perspective that aligns with the optimization of mix design strategies in 3DCP.

## 5. Three-Dimensional Printing of Concrete with Recycled Materials as a Tool for Sustainable Development and Decarbonization of Construction

3D printing technology using concrete mixtures incorporating recycled materials represents one of the most promising directions for the development of sustainable construction. Through the capability of fabricating structures layer by layer directly on-site, while minimizing material losses and ensuring high execution precision, this technology enables a significant reduction in the consumption of primary resources and carbon dioxide emissions. Laboratory studies have shown that the incorporation of secondary raw materials—such as recycled aggregates, waste glass, fly ash, slags, or plastics—not only does not compromise the quality of concrete mixtures used in 3D printing but may in fact enhance their mechanical, rheological, and durability-related properties. It has also been confirmed that the application of geopolymers derived from industrial by-products as partial cement replacements reduces the material’s carbon footprint while simultaneously maintaining or improving its thermal and chemical resistance.

Examples of market-available concrete mixtures incorporating secondary resources, such as Rebetong or ercconcrete, demonstrate that high substitution levels—up to 75% by volume—can be achieved without compromising the required performance parameters (Table 10). At the same time, commercially available low-carbon cementitious solutions, such as ECOPlanet 4B or Vertua Plus, exhibit a carbon footprint reduction of up to 50–66%, further reinforcing the potential of 3D printing as a technology supporting the decarbonization of the construction sector (Table 11). The application of fire-resistant cement-based mixes enables fire resistance levels of up to 1000 °C [204]. Printed structures allow for precise shaping of wall geometries with optimized thermal transmittance coefficients (U-values), which—according to experimental results—can reach values not exceeding 0.15 W/m^2^·K for external partitions [9,205,206]. This indicates that buildings produced by this method are capable of meeting standards typical of energy-efficient housing, while simultaneously reducing the consumption of construction materials by up to 20%. Importantly, the materials used for 3D printing can be sourced locally—from recycling centers, demolition sites, or industrial production facilities—thus decreasing transportation needs and further lowering CO_2_ emissions. This sustainable approach to supply chains enhances the resilience of the construction sector to raw material availability disruptions while also supporting local economies and recycling industries. The use of locally available alternative resources in concrete research (e.g., seawater and sea-sand in GFRP–steel composite tube structures [207]) provides a valuable reference for 3D concrete printing (3DCP), which likewise demands tailored mixtures with a reduced environmental footprint.

The observed development of concrete mixing technologies incorporating recycled materials and their application in 3D printing aligns with European decarbonization strategies and the circular economy model. Research confirms that, thanks to this technology, it is possible to achieve CO_2_ emission reductions of 40–66% compared with conventional solutions, without compromising the quality and durability of the resulting structures.

**Table 10:** Commercially available concrete products incorporating recycled materials [208,209].

Trade Name	Company	Materials	Material of Secondary Raw Materials in 1 m^3^ of the Product (%)	Certificate
Rebetong C20/25	SKANSKA	Portland cement, recycled aggregate, sand, fillers, chemical additives, water	75.5%	Technical and Test Institute for Construction Prague
Rebetong C25/30	SKANSKA	Portland cement recycled aggregate sand, fillers, chemical additives, water	72.3%	Technical and Test Institute for Construction Prague
ercconcrete	ERC-TECH	mix of cement, finely ground recycled brick, ceramics, concrete, microsilica, lightweight artificial aggregate and/or char and/or slag and/or polystyrene and/or at least one organic filler	from 40% to 100%	Technical and Test Institute for Construction Prague

**Table 11:** Commercial cement-based products with reduced CO_2_ emissions [210,211,212].

Trade Name	Company	Materials	CO_2_ Emission Reduction (%)	Certificate
ECOPlanet 4B (CEM IV/B (V) 42.5 N—LH/NA)	LAFARGE	pozzolanic cement, limestone LL ≤0.20% of stone weight, silica fly ash, gypsum/REA-gypsum, dusts from the production of Portland cement, chromium (VI) reducer	up to 40% and 20% recycled material	Building Research Institute (ITB)
Vertua Plus	CEMEX	Portland clinker, limestone, silica fly ash, REA-gypsum, chromium(VI) reducer—iron(II) sulfate	up to 50%	Building Research Institute (ITB)
EcoCrete	Heidelberg Cement	no data	up to 66%	no data

## 6. Conclusions

The conducted literature review clearly indicates that integrating recycled materials into 3D concrete printing (3DCP) is a realistic and effective pathway to reduce environmental impacts without compromising required performance. At the mix level (A1–A3), reported embodied-carbon reductions typically range from ~20–50%, depending on the extent of clinker substitution and the types of secondary constituents; values up to ~48% have been noted when using fine recycled aggregates alongside cement reduction and up to ~62% for mixes incorporating recycled concrete powder after tuning rheology while maintaining printability. Low thermal transmittance of wall elements has also been demonstrated (U ≤ 0.15 W/m^2^·K), together with ~20% material savings enabled by geometry optimization in 3DCP. In addition, commercial low-carbon binder systems have been reported with embodied-carbon reductions on the order of 40–66%. The growing availability of market solutions confirms active development work and technological readiness for deployment.

Despite these advances, industrial-scale implementation requires targeted R&D. First, unified, quantitative rheological protocols specific to 3DCP are needed (e.g., time-dependent yield stress windows, thixotropy, and “open time”) to ensure result comparability and enable controllable printability in mixes with recycled fractions. Second, methods for testing interlayer bond strength and assessing anisotropy (i.e., load-bearing dependence on print direction) must be developed and standardized for recycled-based mixes, and the findings scaled to full-size elements. Third, long-term durability assessments under real service conditions are necessary (carbonation across B1/C3 stages, frost resistance, chloride ingress, fire resistance), accounting for variability of secondary raw materials and QA/QC procedures across the supply chain. Fourth, LCA reporting for 3DCP should be standardized (system boundaries, functional units, explicit accounting for the printing process energy and potential module-D credits) to robustly evidence environmental gains versus conventional technologies. Finally, scale-up studies for mix designs, aimed at achieving stable site-scale production under real field conditions (ambient variability, moisture uptake tolerances of recyclates), are essential to mitigate process risk and ensure repeatable quality of printed components.

Nevertheless, deploying recycled-based 3DCP is a clear step toward sustainable development and, in the long term, aligns with the objectives of the European Green Deal and global climate-neutrality strategies by 2050. The integration of secondary raw materials into additive construction processes can become a cornerstone of the sector’s transition toward a circular economy and a low-carbon built environment.

## Figures and Tables

**Table 2:** Physical, chemical, and functional characteristics of fly ash vs. bottom ash [56].

Parameter	Fly Ash (FA)	Coal Bottom Ash (CBA)
Source	Particles from flue gas transport	Clinker-like lumps from boiler bottom
Density (g/cm^3^)	2.18–2.32	2.20–2.40
Moisture content (%)	0.01–0.11	26.5–50.1
Median particle size (mm, d50)	0.01–0.03	0.42–2.19
Sand fraction (%)	3.5–7.5	39.8–56.9
Dust fraction (%)	77.6–90.2	5.3–18.0
SiO_2_ (%)	51.1–64.4	52.0–64.9
Al_2_O_3_ (%)	19.4–27.9	17.9–24.7
Fe_2_O_3_ (%)	3.5–8.0	4.9–9.6
CaO (%)	1.3–6.2	2.1–7.1
SO_3_ (%)	0–2.1	0–1.3
pH	8.1–12.8	8.3–9.7
Loss on ignition (%)	0.9–2.1	-
Practical properties	Improves workability, durability, increases compactness	Good permeability, water retention, mechanical stability

**Table 5:** Summary of recycled constituents and industrial by-products for 3D concrete printing (3DCP): process-relevant advantages and disadvantages.

Material	Advantages (3DCP)	Disadvantages (3DCP)	References
Fly ash (FA)	Lowers plastic viscosity and often reduces τ_0_(dyn) → smoother pumping/extrusion; can stabilize buildability at moderate dosages; tends to widen the printability window.	High dosages may delay setting and reduce early green strength; buildability can drop without viscosity control/admixtures.	[154,155]
Ground granulated blast-furnace slag (GGBFS)	In alkali-activated materials/geopolymers (AAM/GP) systems: strong structural build-up (thixotropy) → high buildability; in cement blends with FA: can keep extrusion stable at lower GWP.	Activator sensitivity (Na/K, Si/Al, temperature) → risk of too short printability window or nozzle clogging if poorly tuned.	[47,156]
Silica fume (SF)	Strong increase of τ_0_(stat) and early stiffness → improved buildability and filament shape retention; can mitigate anisotropy via cleaner bead geometry.	Excess dosage raises viscosity → pumpability issues and potential nozzle blocking; requires precise W/B and SP control.	[157,158]
Recycled glass powder (WGP/GWG)	At small–moderate dosages: better matrix packing, longer printability window, and stable buildability in printable mortars.	Very high replacements may shorten open time (in GP/AAM) and degrade filament quality without viscosity tuning.	[100]
Recycled fine aggregate (RFA)	At modest levels can maintain extrusion; with concurrent cement reduction may keep buildability and extend printability by matrix tuning.	Higher fractions increase water/admixture demand, may raise τ_0_(dyn) and shorten open time; green strength often drops.	[32,159]
Recycled brick aggregate (RBA)	Rough angular surface may enhance interlayer mechanical interlock; feasible sand replacement with controlled grading/moisture.	High absorption/roughness → higher water demand and ↑ τ_0_(dyn); can reduce filament stability without paste adjustments.	[118]
Steel slag aggregate (SSA/SA)	Good extrudability at tuned SA/C and W/C; rough texture helps bead stability; synergy with SF improves buildability.	Higher absorption → water/admixture corrections needed; buildability moderate without viscosity modifiers.	[126,160]
Rubber aggregates (crumb rubber, GWTR)	Lighter mixes with damping; can keep extrusion continuity after surface treatment and paste tuning.	Strength reduction and shorter open time if untreated; viscosity ↑ → pumpability must be re-balanced; anisotropy may intensify.	[124,161]
Geopolymers (AAM/GP based on FA/GGBFS)	High buildability with fast structural build-up; early green strength enables layer stacking at low deformation; stable extrusion when τ_0_/viscosity sits in the process window.	High sensitivity to activator chemistry and curing temperature → risk of too short printability or pumpability loss; requires tight process control.	[156,162]

## Data Availability

No new data were created or analyzed in this study. Data sharing is not applicable to this article.
